# Rehabilitation of Post-traumatic Anterior Maxillary Osseous Deficit Using Iliac Onlay Bone Graft Combined With Dental Implants

**DOI:** 10.7759/cureus.37188

**Published:** 2023-04-06

**Authors:** Ananya Mittal, Shandilya Ramanojam, Saurabh Khandelwal, Mohamed Umer Valiulla

**Affiliations:** 1 Oral and Maxillofacial Surgery, Bharati Vidyapeeth Dental College and Hospital, Pune, IND

**Keywords:** post-traumatic defect, autologous graft, dental implant, oral augmentation, iliac crest graft

## Abstract

Fracture of the anterior maxilla usually causes a scooped-out defect in this region which leads to loss of lip support and a sub-optimal condition for placement of implants. The iliac crest is a frequently used donor location in oral and maxillofacial procedures for bone augmentation in order to restore jaw deformities brought on by trauma or pathological diseases prior to the placement of dental implants. Here we present the case of a patient who had undergone reconstruction of the maxillary osseous defect caused due to trauma by iliac crest grafting, followed by placement of dental implants after six months.

## Introduction

Dental implants have evolved over the past ten years into a standard procedure for the rehabilitation of patients who are partially or completely edentulous [1]. For the retention and durability of implants as well as for positive results, there must be enough residual bone volume. Yet, in some circumstances, unfavourable local conditions such as jaw degeneration, bone defects brought on by different types of osteomyelitis, and post-traumatic sequelae may leave less bone volume for implant insertion as a result of flaws in one or more dimensions. [2]. 

The iliac crest is a frequently used donor location in oral and maxillofacial surgeries to harvest bone for reconstruction before placement of dental implant or to correct jaw deformities caused due to trauma or various clinical diseases [3]. The harvested bone from the anterior iliac crest has exceptional osteogenic, osteoconductive, and osteoinductive properties, making it well-established donor site [4,5]. There are three popular techniques for harvesting an autogenous bone graft from the anterior iliac crest. The first is called trephine curettage [6], which is a technique for obtaining cancellous bone from either the anterior or posterior ilium. The second method is the trapdoor method of harvesting bone graft for which the anterior ilium is the most suited site. The third technique is the a sub-crestal window technique, which is used to harvest bone graft in a block of any size or shape [7]. The iliac crest is the frequently preferred donor site over calvaria or tibia bone grafts when the volume of bone that may be harvested intraorally is limited [8-10]. Generally, bone grafts for oral augmentation are taken from the anterior region of the iliac crest, where up to 26.29 mL of cortico-cancellous graft can be acquired [11].

Here we have described the case of a patient who had undergone a two-part procedure. First, a bone from the anterior iliac crest was harvested to augment the deficient anterior maxilla following trauma, followed by the secondary placement of implants. There is a dearth of literature regarding the use of iliac crest graft for the regeneration of post-traumatic bone defects. This article aims to highlight this technique of augmentation of bone for implant placement.

## Case presentation

**Figure 1:**
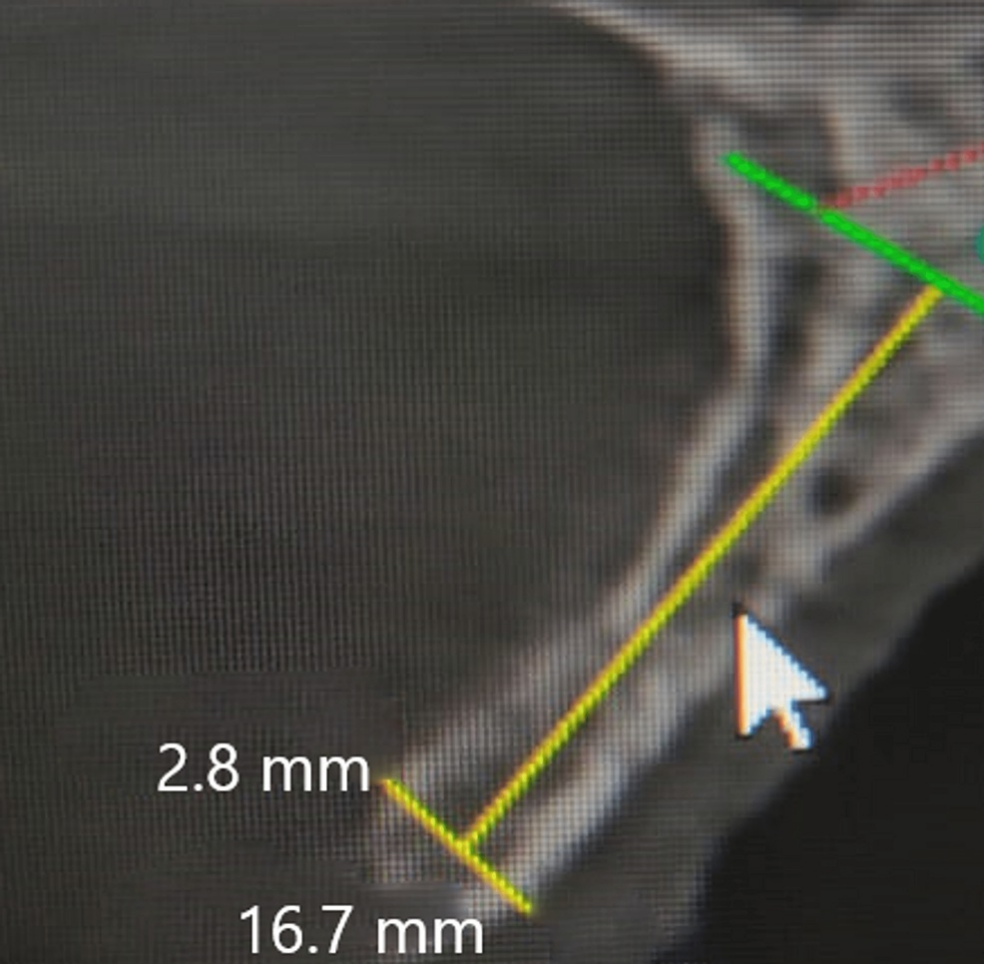
Preoperative CBCT of the patient CBCT: Cone-beam computed tomography

**Figure 2:**
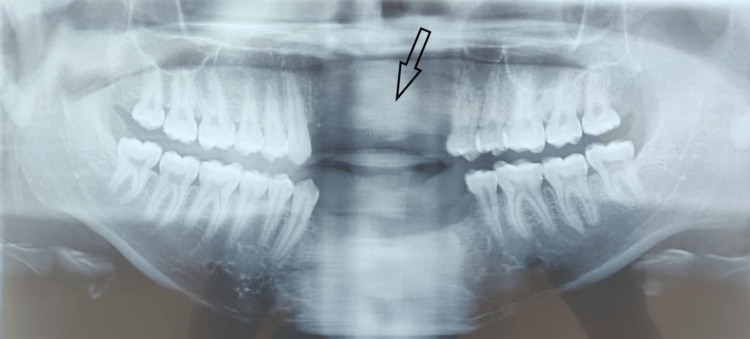
Preoperative radiograph after eight months of road traffic accident

An 18-year-old male came to our institute with the complaint of missing front teeth following a road traffic accident eight months back. The surrounding soft tissues appeared healthy with no inflammation. An orthopantomogram (OPG) and cone-beam computed tomography (CBCT) were done, which revealed excessive bone loss in the maxillary anterior region, which was insufficient for implant placement (Figures 1, 2). The width available was 2.8 mm and the height was 16.7 mm. A two stage surgical procedure, augmentation of the anterior maxilla using autologous graft from anterior iliac crest followed by implant placement in the same region after six months was planned. Both the surgical stages were performed by the same surgical team. A written informed consent was taken by the patient for all the surgical procedures.

**Figure 3:**
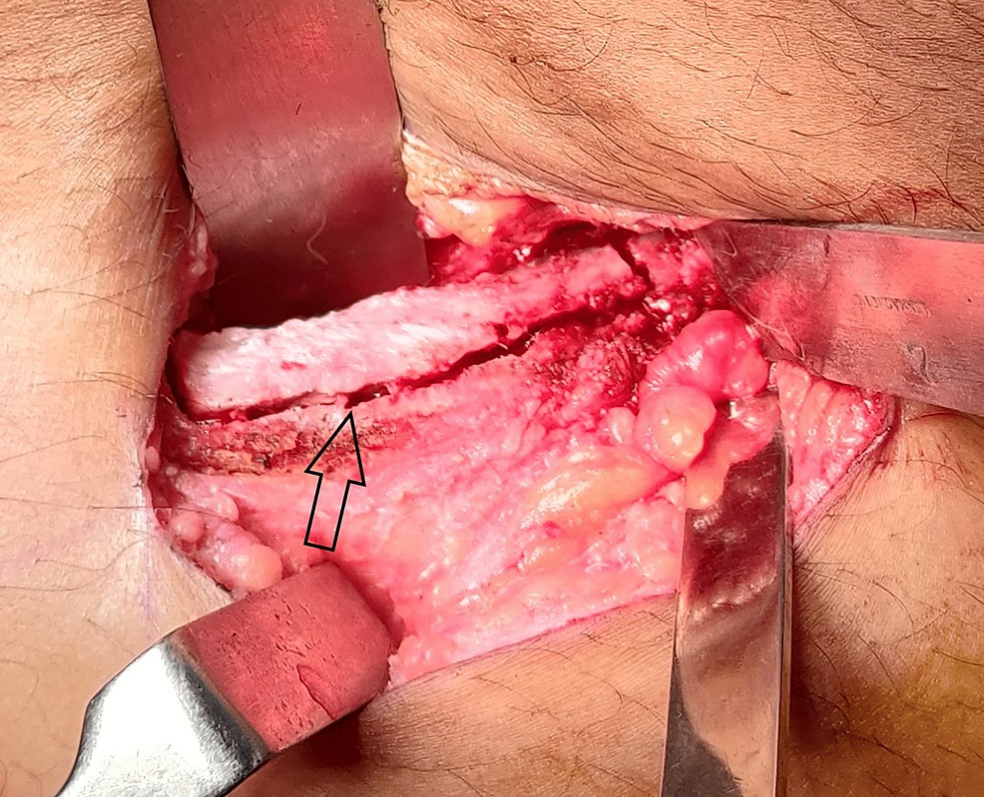
Monocortical autonegonus bone graft harvesting procedure from the anterior iliac crest

Bone augmentation by iliac crest graft was planned under general anesthesia. All aseptic protocols were followed. A skin incision was made along the anterior iliac crest, preserving the surrounding nerves. Subperiosteal dissection of the overlying soft tissues was carried out to expose the medial and lateral surfaces of the iliac crest. The outline of the bone block to be harvested was made using a straight handpiece and bur (Figure 3). Chisel and mallet were then used to harvest a monocortical (medial cortex with cancellous part) autogenous graft measuring approximately 5x2x3 cm from the donor site. The sharp bone edges were smoothened and a mini-vac drain was placed at the site of the anterior iliac crest. The suturing was done in layers, starting with the periosteum of the anterior iliac crest, followed by the muscle attachment and subcutaneous layer using 3-0 resorbable polyglactin suture. The skin was sutured using a 3-0 non-resorbable suture.

**Figure 4:**
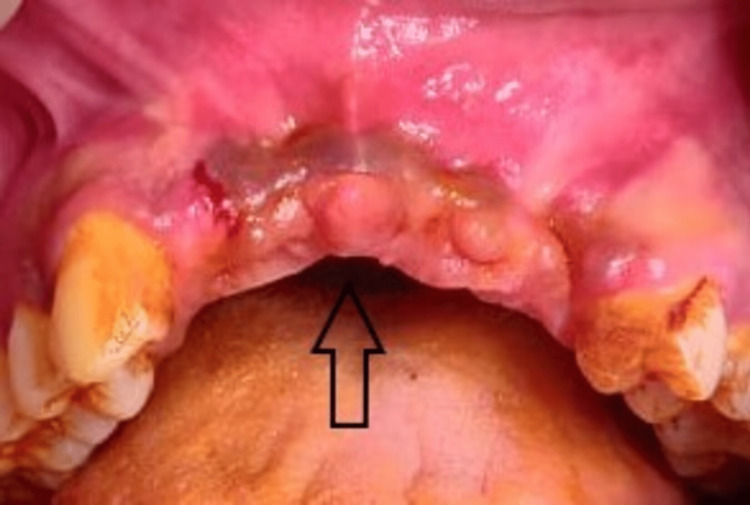
Preoperative recipient site

**Figure 5:**
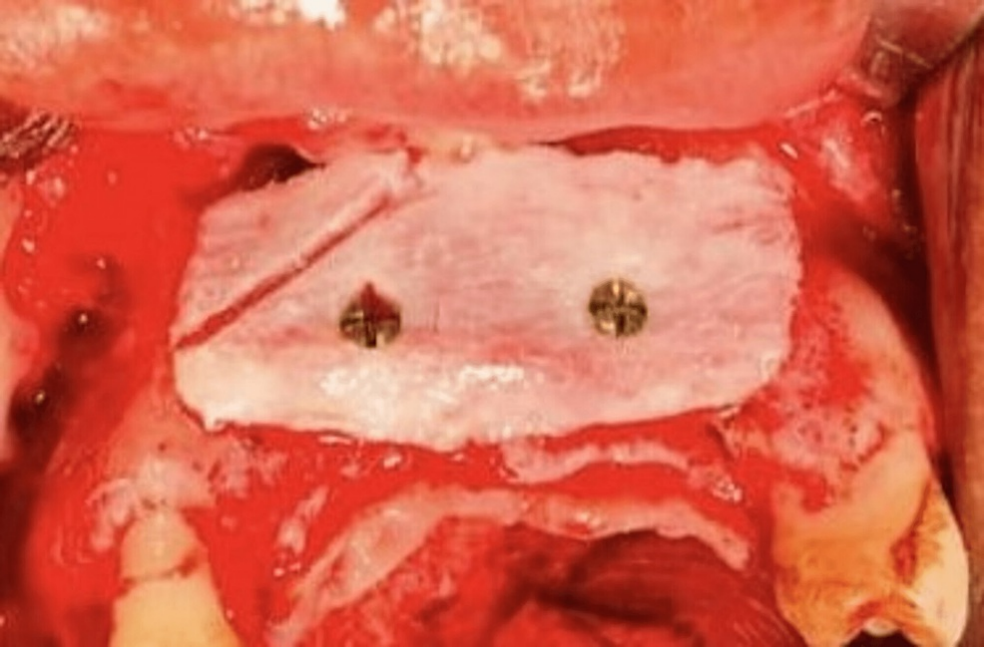
Iliac crest graft fixed to the deficient anterior edentulous site using two titanium mini-screws

A mucoperiosteal flap was elevated in the recipient site and the recipient bed was prepared (Figure 4). The harvested iliac bone block was sculpted according to the size and shape of the recipient site and was fixed to the basal bone using titanium mini-screws (Figure 5). The cancellous part of the graft faced the native bone. The iliac crest graft was oversized to compensate for the bone resorption during the postoperative period. The gaps between the graft and the recipient bone were filled using cancellous bone obtained from the donor site. Tension-free closure of the flap was achieved using 3-0 resorbable polyglactin suture. The postoperative period was uneventful and the patient was discharged on the third postoperative day after removal of the mini-vac drain. Suture removal at the donor site was done after 10 days. There were no intraoperative or postoperative complications.

**Figure 6:**
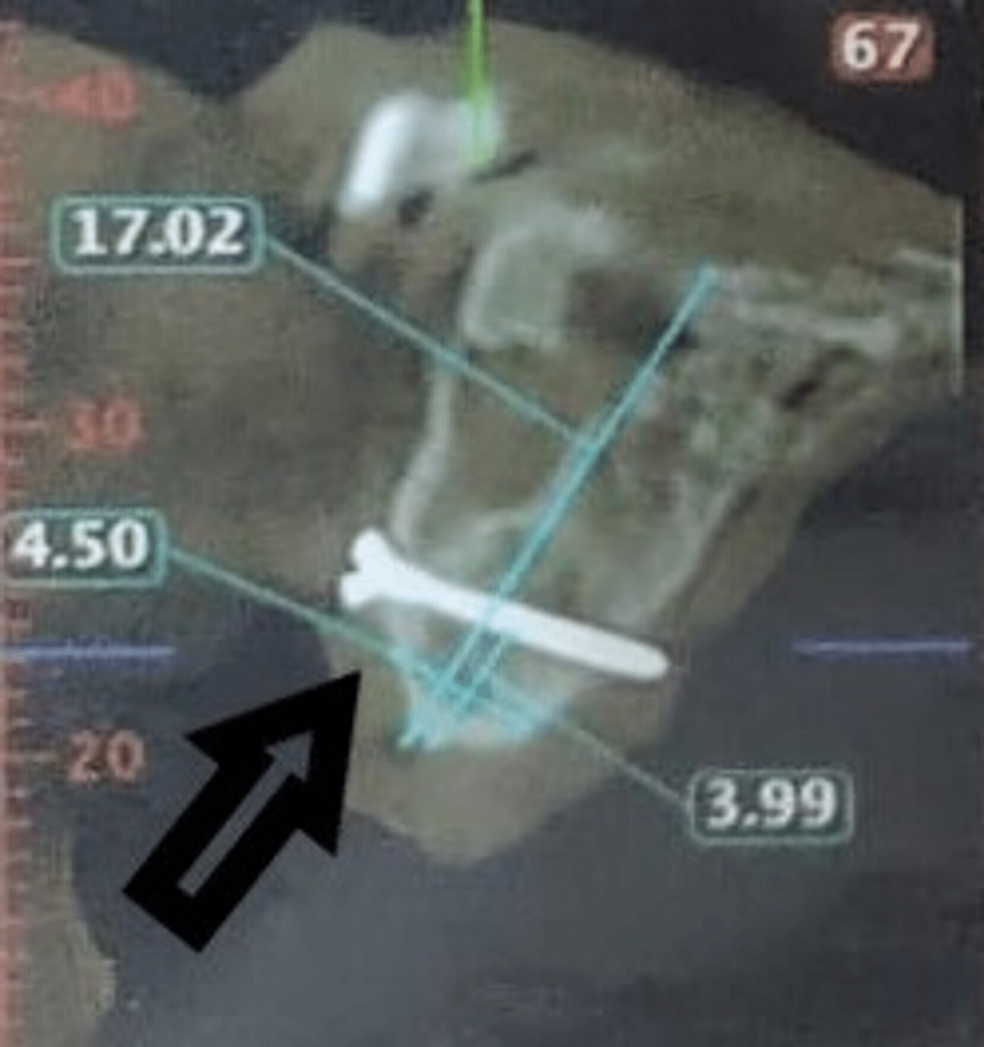
Postoperative CBCT of the patient CBCT: Cone-beam computed tomography

**Figure 7:**
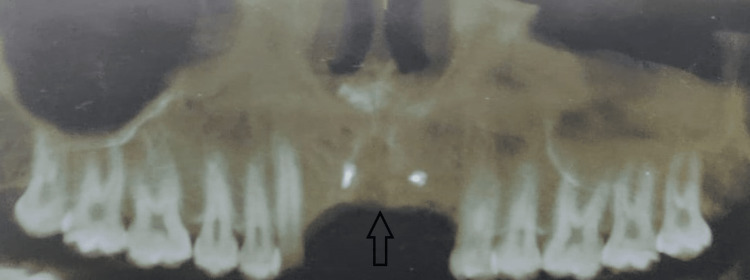
Postoperative CBCT after six months of the first surgical procedure CBCT: Cone-beam computed tomography

The patient was kept under regular follow up. After six months of the first surgery, implant placement was planned in the maxillary anterior region. A cone beam computed tomography (CBCT) scan was done to assess the amount of bone available at the recipient site (Figures 6, 7). The width available was 4.5 mm and the height was 17.02 mm after six months of the first surgery.

**Figure 8:**
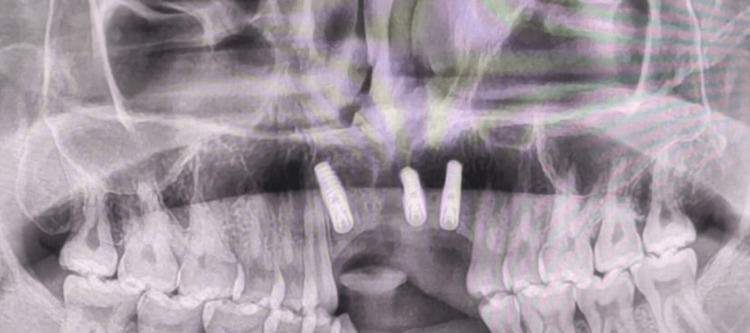
Postoperative radiograph after implant placement

The amount of bone gained from the augmentation procedure at the implant site acted as a reference for the choice of implant length and diameter. The preoperative width of the bone in the edentulous region was 2.8 mm as compared to 4.5 mm after six months of surgery. The preoperative height of the bone at the edentulous region was 16.7 mm as compared to 17.02 mm postoperatively after six months. A full-thickness flap was elevated for conventional implant placement. Then, the titanium mini-screws which were used to fix the graft were removed. A total of three implants were placed in the maxillary edentulous region, measuring 3.5x11.5 mm, 3.5x10 mm and 4x11.5 mm (Figure 8). The implants engaged the native as well as the harvested bone so that they had a complete cortical coverage from all sides and good primary stability could be achieved. Torque of 40 Ncm was achieved after placement of each of the three implants. The flap was closed using non-resorbable silk sutures and suture removal was done after seven days. Four months after the dental implants had been osseointegrated, the patient was given implant-supported fixed prosthesis.

## Discussion

Autogenous bone graft is considered the ‘gold standard’ for pre-prosthetic augmentation procedures [12]. Intraoral sources are the common sites for harvesting bone graft for correction of small osseous defects. However, in this case, the osseous defect was comparatively bigger in size. So an extraoral donor site was chosen and it was decided to harvest the bone graft from the iliac crest. In order to reconstruct large bone defects and place osseointegrated dental implants for total aesthetic and functional rehabilitation, the iliac crest is the most commonly used extraoral donor site as it offers sufficient amount of cortical and cancellous bone [13]. In this case, we used the anterior iliac crest for bone harvesting. The posterior iliac crest was not used due to the need to turn the patient over, preventing concurrent harvesting and graft placement. The scaffold provided by the corticocancellous block graft will eventually be almost entirely resorbed and replaced by new bone. This remodeling process is referred to as creeping bone substitution [14]. For this process to proceed unobstructed, corticocancellous graft must be rigidly fixed to the recipient location. Even slight movement at the point where the graft meets the recipient site could endanger capillary ingrowth from the recipient bed, leading to avascular necrosis and graft loss. To avoid any micromovement, it is always desirable to attach the bone graft with at least two screws [15], as was done in this case.

According to some studies, the survival rate of implants after augmentation using iliac bone grafts has been reported to be 92.4% after a follow-up period of five years [16]. Despite their benefits, iliac bone grafts are linked to high levels of bone resorption, which is greatest during the early healing phase [17]. Other complications which may occur at the donor site include infection, fracture, hematoma, neural injuries, vascular injuries, hernia, chronic pain and poor cosmetic result. It is still debatable whether the placement of implants should be done right away after graft placement or deferred for a few months following bone grafting. The various complications which may occur at the intraoral recipient site include secondary infection, graft failure, graft exposure, etc. The majority of earlier research found that the two-stage method had better results than the one-stage technique [18]. The hypotheses for this is that the implant will integrate more favourably and steadily following a period of bone healing and revascularization [2]. One-stage surgery is preferred, according to some other authors, because it requires fewer surgical procedures and heals more quickly [19]. However, reports of the one-stage approach's high and unpredictable rates of bone resorption have also been made [20], which can result in poor primary implant stability and poor prosthesis orientation. In this case, we used a two-stage approach based on the previous studies.

## Conclusions

To conclude, patients can safely undergo the surgery of harvesting bone grafts from the anterior iliac crest for correction of large intraoral osseous defects. While some postoperative morbidity at the donor site may occur, their frequency is minimal and they typically last only for a brief time. Severe issues are quite uncommon. Currently, rehabilitation in individuals with severely resorbed alveolar crests, bone deformities from trauma, and congenital anomalies is often done with the goal of restoring their masticatory function and aesthetic appeal. A variety of cases have been documented related to the use of iliac crest graft for the reconstruction of osseous defects. However, very few cases have been reported in which the iliac crest graft was used to correct post-traumatic osseous defects. In this case, it was demonstrated that the iliac crest could be used successfully as an extraoral donor site for intraoral bone augmentation. In patients with insufficient or severely atrophied bone, endosseous implants and the use of bone grafts will enhance prosthetic rehabilitation. Thus, combining iliac crest bone graft and dental implant systems can result in adequate long-term repair in patients with atrophic jaws.
